# Characteristics of drought vulnerability for maize in the eastern part of Northwest China

**DOI:** 10.1038/s41598-018-37362-4

**Published:** 2019-01-30

**Authors:** Ying Wang, Wen Zhao, Qiang Zhang, Yu-bi Yao

**Affiliations:** Key Laboratory of Arid Climatic Change and Reducing Disaster of Gansu Province, Key Open Laboratory of Arid Change and Disaster Reduction of CMA, Institute of Arid Meteorology, China Meteorological Administration, Lanzhou, Gansu, 730020 China

## Abstract

Based on information distribution and diffusion method theory and combined with the standardized precipitation index and relative meteorological yield data, meteorological factors and social factors were comprehensively considered to assess the vulnerability of maize (*Zea mays*) to drought. The probability distribution curve of meteorological drought degree (MDD) and relative meteorological yield in the eastern part of Northwest China (Gansu, Ningxia and Shaanxi) from 1978 to 2016 were obtained, using a two-dimensional normal information diffusion method to construct the vulnerability relationship between MDD and relative meteorological yield. The drought vulnerability curve of maize in the study area was obtained. The probability distribution of MDD was multiplied by the fragility curve and summed to obtain the multi-year average risk. The MDD probability distribution curve showed that the probability of moderate drought in Shaanxi was relatively high, followed by Gansu and Ningxia. The probability distribution of Gansu was more discrete. The probability of strong meteorological drought in Ningxia was high, followed by Shaanxi and Gansu. Probability distribution of relative meteorological yield for maize in Gansu Province was highly discrete, with thick tailings, large uncertainties, and more extreme values, which were strongly affected by meteorological conditions, followed by Shaanxi and Ningxia. Taking meteorological drought as the cause and maize damage as the result, the vulnerability relationship between MDD and drought damage was obtained. With an increased MDD, the relative meteorological yield of maize gradually declined. From the average value, when MDD was less than −2.60, the relative meteorological yield of maize was reduced within 15%; when MDD was greater than −2.60, the relative meteorological yield of maize increased within 10%. When the degree of meteorological drought exceeded −2.2, maize was most vulnerable to drought in Shaanxi followed by Ningxia and Gansu. When meteorological drought was less than −2.2, maize was most vulnerable to drought in Shaanxi followed by Gansu and Ningxia. The expected values of relative meteorological production in Gansu, Ningxia, and Shaanxi were 1.36%, 2.48%, and −1.76%, respectively; therefore, Shaanxi had the highest maize drought risk, followed by Gansu and Ningxia. This research had a clear physical background and clear risk connotations. The results provide a data foundation and a theoretical basis for drought disaster reduction for maize in the study area.

## Introduction

Most of China lies in the East Asian monsoon climate area, which has frequent droughts^1^. Chinese agricultural statistics^2–10^ show that the five major meteorological disasters (drought, flood, wind hail, frost, and typhoon) occurred between 1978 and 2016; the annual average area affected by drought was 22,676 kha, accounting for 53% of the total meteorological disaster area in China. Drought during this period reduced total grain output by more than 4.7%. Therefore, drought is the most serious meteorological disaster affecting agriculture in China. Northwest China extends into Eurasia and more than 80% of the area is arid or semi-arid because humid oceanic air is blocked by the Qinhai-Tibet plateau in the southwest. Since the 1970s, climate warming has increased atmospheric water-holding capacity and changed the atmospheric circulation pattern, giving Northwest China a clear warming and drying trend. Although there are local warm and wet phenomena, overall drought and continuous drought trends have increased^11–13^, and let to increased drought risk.

Maize (*Zea mays*) is one of the main grain crops in Northwest China. It represents >30% of the total planting area of grain crops, and provides >50% of the region’s total grain yield^14^; thus, maize production plays a critical role in the regional agricultural economy. According to the physiological characteristics of maize, accumulated temperature meets its growth requirements, but precipitation is the main factor influencing its yield and so meteorological drought leads to large fluctuations in maize yield and could lead to a food security crisis^15^. Under such circumstances, managing maize drought risk in Northwest China has become urgent. Related research has concentrated on meteorological elements and the damage to maize, such as causes of drought^16,17^, spatial and temporal distribution characteristics of meteorological drought^18–21^, influence of drought^22,23^, and drought damage and risk assessment of maize^24–27^. However, the relationship between meteorological drought and maize damage has not been determined, that is, the vulnerability between drought and disaster. Mastering such relationships is important for drought risk management of maize.

In the Second Working Group of the Fifth Assessment Report of the Intergovernmental Panel on Climate Change, vulnerability is defined as sensitivity or susceptibility to harm and lack of capacity to cope and adapt^28^. The advantages of this concept are reflected in the integration of risk sources, risk bearing bodies, and different space–time dimensions that act on the receiver. Specific research on drought vulnerability at the complex level mainly adopts the index system evaluation method; i.e., establishing a multi-objective evaluation index based on disaster composition factors; assigning different weights, and finally establishing an evaluation model^29,30^. This method focuses on the natural attributes of drought vulnerability, with a sufficient physical basis to facilitate qualitative analysis^31–34^. The crop growth model produces rate of yield loss under different scenarios, based on meteorological data, soil physicochemical properties, and agricultural cultivation strategies^35^. This method predicts or simulates disaster and is not a risk analysis. The traditional probability estimation and regression model can analyze historical drought data and relevant meteorological data, determine the type of fitting distribution, and then assess risk in the study area^36,37^. However, this method can only be used to analyze deterministic relationships, or to describe the standard form in a statistical sense. Due to the complexity of natural disaster system, it is difficult to assume the appropriate probability distribution type, and so non-parametric estimation methods are important. The histogram method is the simplest non-parametric estimation method for probability distribution because it ignores the difference between different sample points falling within the same histogram interval. The theory of information distribution and diffusion allows the utilization of this information and does not require any assumptions about the type of overall probability density function to improve estimation accuracy^38^. The difficulty in the assessment of natural disaster vulnerability is not only to give a more reliable overall probability distribution with limited information, but more importantly, the information distribution and diffusion method provides a new way to understand the structure of given sample information. In addition, in assessment of natural disaster vulnerability, it is necessary to study the relationship between the possible destructive power of a disaster and the degree of damage to the disaster-bearing body. At this point, the information distribution and diffusion method can be used to construct causal fuzzy relationships^39,40^. In view of this, the information distribution and diffusion method has been used in disaster vulnerability research. For example, Huang *et al*.^41^ used this method to study the vulnerability relationship between annual precipitation and the proportion of the affected population. Wang *et al*.^42^ analyzed the characteristics of drought vulnerability in northeast China based on meteorological and social factors. For historical reasons, data on drought disaster and maize yield in Northwest China are discontinuous and of short time series. Therefore, based on the information distribution and diffusion method, this paper uses meteorological drought as a hazard (risk sources) and relative meteorological yield of maize (risk result). The relationship between the two can be defined as the vulnerability function of maize to drought, and can be used to analyze the characteristics of drought vulnerability of maize in Northwest China. The research results can provide basic data and theoretical support for drought risk management of maize production in Northwest China.

## Study Area

Northwest China includes five provinces: Gansu, Ningxia, Shaanxi, Qinghai, and Xinjiang. Geographically, the region is in the Eurasia hinterland, surrounded by high mountains which prevent access to ocean water vapor; therefore, drought disasters occur periodically. The area has complex terrain, a wide range of altitudes, and mainly comprises plateaus, mountains, and basins. Soil types are diverse, including chestnut, gray cinnamon, loess, and black soils. Soil organic matter is strongly mineralized and, generally, there is low organic matter content. The region has a continental monsoon climate with abundant sunshine, strong evaporation, large daily and annual temperature differences, and frequent disastrous weather such as drought. The climatic conditions of the study area are shown in Table 1. Maize is the main food crop in Gansu, Ningxia, and Shaanxi, and its average annual output accounts for 41%, 34%, and 41% of total grain output, respectively.

**Table 1:** Climatic conditions in the study area.

Province	Average annual precipitation (mm)	Average annual temperature (°C)	Average annual water surface evaporation (mm)
Gansu	300	8.1	1000–3500
Ningxia	450	8.6	800–1600
Shaanxi	600	13.0	1100–2500

**Figure 1:**
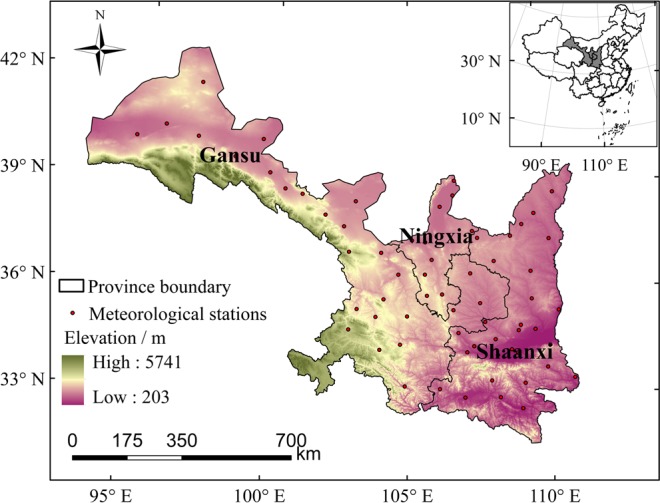
Location of the study area (inset map shows China).

Qinghai has an average elevation of 4000 m above sea level and accumulated temperature in most areas cannot meet the needs of maize growth. Maize is planted only in small areas in the valleys of eastern Qinghai Province. Annual precipitation in Xinjiang is 200 mm, and crop growth depends on glacier and snow melt water. Therefore, these two regions were not considered in studying the vulnerability relationship between meteorological drought and maize production (Fig. 1).

## Data and Methods

### Data sources

According to the purposes of this maize drought vulnerability research in the study area, the main data selected were, as follow:

1. Meteorological data: monthly precipitation data (mm) between 1978 and 2016 were obtained from 66 meteorological stations in the study area, made available by the National Meteorological Science Data Sharing Service Platform (http://data.cma.cn/). The data are standardized, quality controlled, and have been widely applied in business and research work.
2. Maize yield data: the maize unit yield (kg/hm^2^) was derived from the “Statistical Data of New China Agriculture for 60 years” for the years 1978 to 2008 and from “China’s Agricultural Statistical Report” for the years 2009–2016. These data came from the Chinese Ministry of Agriculture, which uses unified and standard statistical methods to report agricultural yields; their data are widely used in disaster research in China^37^.

### Research methods

#### Degree of regional meteorological drought

The standardized precipitation index (SPI) based on precipitation data was selected as the meteorological drought index. This index has many advantages such as simple calculation, good stability, multiple time scales, and space–time comparability. It has been widely used in drought monitoring^43,44^ and can be used to accurately reflect the meteorological drought characteristics in Northwest China^45–48^. SPI assumes that changes in precipitation follow a gamma distribution and it uses mathematical methods to convert the cumulative frequency distribution of precipitation into a standard normal distribution. The multi time-scale advantage of SPI can not only reflect the change of precipitation at a short time-scale, but also the evolution of water resources at a long time-scale. The specific calculation methods and time scales have been previously described^49–51^ 3-month time-scale SPI (SPI3) represents moisture profit-and-loss on a short time-scale and it is commonly used in agricultural drought studies^46^. Therefore, SPI3 could be calculated as a hazard factor for maize drought in all sites in the study area. Since the main growth period of maize is April–September, SPI3 values from April 1978 to September 2016 were considered. Because the spatial distribution of meteorological stations in various provinces was relatively uniform (Fig. 1), the SPI3 values from all meteorological stations in each province were regionally averaged (Equation 1), and the results were used as regional meteorological drought indicators in the province to calculate a spatial statistical scale of maize production.

1 $$X=\sum _{i=1}^{n}{x}_{i}/n$$

where *x*_*i*_ is the SPI3 for each site in the province, and *n* is the number of sites in the province.

**Figure 2:**
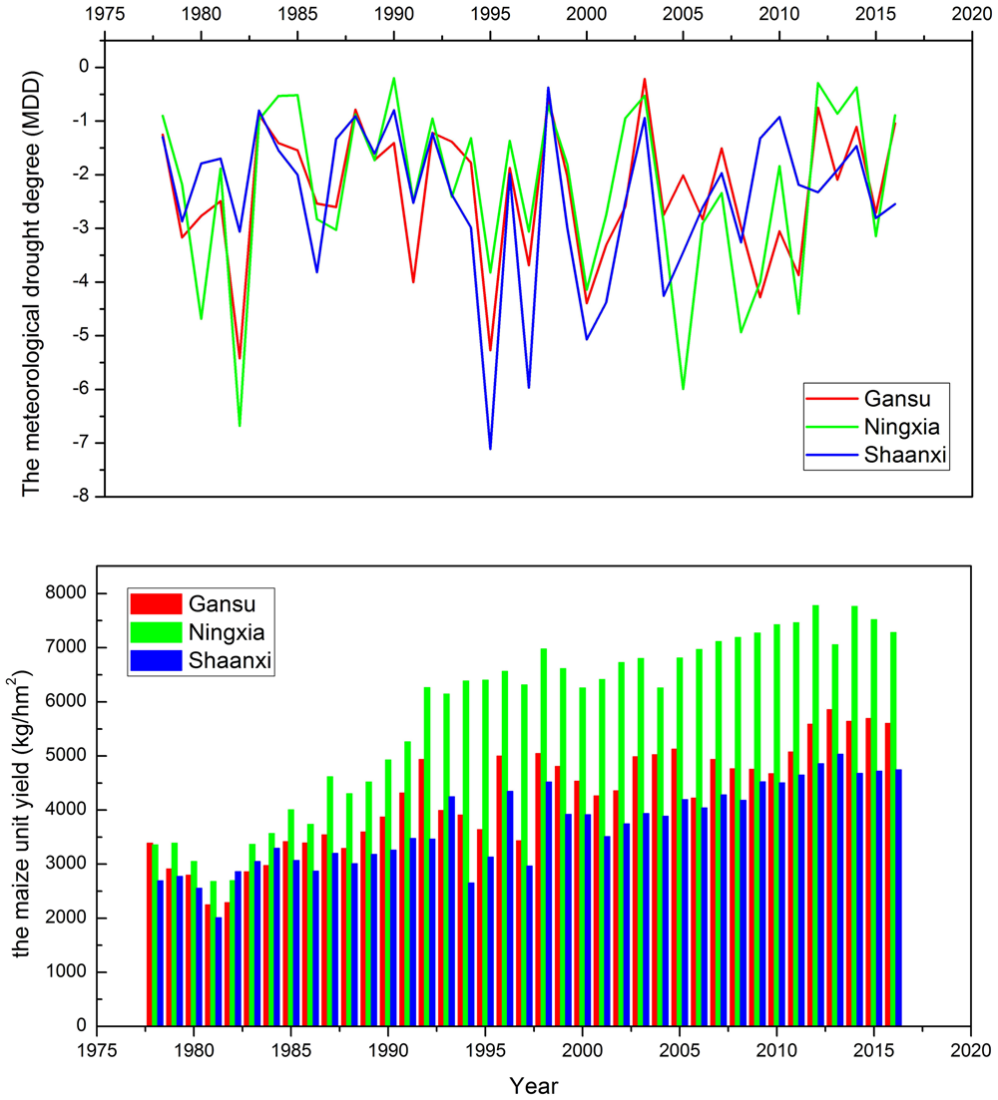
The interannual variability of annual MDD and average maize unit yield in the study area.

The meteorological drought degree (MDD) was determined by the intensity of drought and its time length, given by Equation (2):

2 $$MDD=\sum _{i=1}^{D}{X}_{i}$$

where *X* is the regional drought indicator for each month in the growth period, which represents drought intensity; *D* is the month when the SPI3 is lower than the value *S* in the year; that is, the time duration of drought in the growth period. According to the normal distribution curve of SPI^51^ (Table 2), the threshold of SPI drought classification was obtained, and *S* = 0. The interannual variability of annual MDD and average maize unit yield in the study area are shown in Fig. 2.

**Table 2:** Classification standard of drought based on the standardized precipitation (SPI) index.

Class	Drought type	SPI value
1	Light	−1.0 < SPI ≤ 0
2	Medium	−1.5 < SPI ≤ −1.0
3	Heavy	−2.0 < SPI ≤ −1.5
4	Special	SPI ≤ −2.0

#### Separation of relative meteorological production

Maize yield can be categorized into three yield types: meteorological, trend, and random errors. The meteorological yield refers to yield components that are mainly affected by a short period change in meteorological factors^52^. The trend yield can be regarded as a long-period yield component that reflects the changes in productivity in a region over an historical period, such as soil properties, species characteristics, agricultural policies, and science and technology. The random errors are generally neglected. This study used the method of linear moving average to separate trend yield^53,54^ and obtain the relative meteorological yield.

3 $${Y}_{{\rm{w}}}=Y-{Y}_{{\rm{t}}}$$

4 $${Y}_{{\rm{r}}}={{Y}}_{{W}}/{Y}_{t}\times 100 \% $$

where *Y* is actual yield, *Y*_*t*_ is trend yield, *Y*_*w*_ is meteorological yield, and *Y*_*r*_ is relative meteorological yield (%). When actual yield is lower than the trend yield, *Y*_*r*_ is negative and it is called the yield reduction rate; in contrast, when actual yield exceeds the trend yield, *Y*_*r*_ is positive and is called the yield increase rate. The relative meteorological yield showed that the amplitude of grain yield fluctuation was not limited by time and region, and was comparable and so can better reflect the impact of meteorological factors on yield, and is widely used in agricultural drought risk research^52,55^. For specific parameters calculation, please see Appendix A.

#### The information and diffusion distribution method

When dealing with small amounts of sample data, because the information contained is not complete enough to accurately determine the statistical law that the sample should follow, the method of information distribution and diffusion is proposed^39,56^. The information distribution method is the optimization of sample probability calculated by the traditional histogram method, in which histogram boundary fuzzification is used to make available information on the transition boundary, so that the probability using a small sample is more reasonable^56^ (see Appendix B). The information diffusion method is based on the evolution of molecular diffusion theory, which allows the samples to diffuse normally and obtain sufficient sample size for research (see Appendix C).

#### Drought risk model

In risk analysis, the *R* = *H*°*D* model is widely used^56^, where *R* is the risk, *H* is the function family that describes the source of the risk, *D* is the function family of vulnerability that describes the risk bearer body, and “°” is the synthesis rule family. When the connotation of the risk is the expected value of the relative meteorological yield, the index system describing it is composed of two indicators: relative meteorological yield (*D*) and the probability (*H*), and “°” can be a multiplication operation^56^.

If *R* is the maize drought risk, *p*(*x*) is the probability distribution function of MDD, and *F*(*x*) is the drought vulnerability function of maize:

5 $$R={\int }^{}P(x)\cdot F(x)dx,$$

*P*(*x*) is estimated by the MDD in history, and *F*(*x*) is estimated by the relative meteorological yield of maize. When the probability distribution is discrete, it can be expressed as:

6 $$R=\sum _{j=1}^{m}P({u}_{j})\cdot F({u}_{j})$$

Studies have shown that information distribution and diffusion methods can greatly improve the utilization of incomplete information and significantly improve the accuracy of system identification^57^. Therefore, *P*(*x*) and *F*(*x*), estimated by this method, were used to calculate the risk values; that is, the uncertainty of meteorological factors was taken into consideration, as was the nonlinearity of the vulnerability curve. The results of such analyses are more consistent with objective reality.

## Results and Analysis

### Probability distribution estimation of MDD

According to the definition of the intensity and the time length for drought in 3.2.1, Equations (1) and (2) were used to obtain a sample of the MDD in maize growing seasons in Gansu, Ningxia, and Shaanxi between 1978 and 2016. Using Gansu as an example, Please see Appendix D.

**Figure 3:**
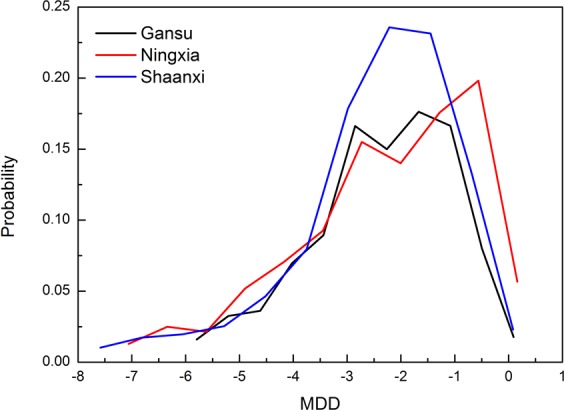
Probability of meteorological drought degree (MDD).

The probability distribution curve (Fig. 3) showed two probability peaks in Gansu, which occurred at the MDD of −1.67 and −2.85, with corresponding probability values of 0.18 and 0.17; Ningxia also had two probability peaks, at MDD of −0.56 and −2.73, with corresponding probability values of 0.20 and 0.16; Shaanxi had one probability peak at the MDD of −2.22, with a probability value of 0.24. The skewness of probability curves in Gansu, Ningxia, and Shaanxi was 0.24, 0.43, and 0.81, respectively; and the corresponding kurtosis of probability curves in Gansu, Ningxia and Shaanxi was −1.76, −1.37 and −1.07. These data show that the probability curves of the MDD in the three areas were all right-biased and data distributions were flatter than the standard normal distribution. The probability curve in Shaanxi had the largest degree of right-bias and kurtosis, indicating a relatively high probability of moderate drought, followed by Gansu and Ningxia. The distribution of probability curves in Gansu was the most flat and data dispersion was strong. The trailing curve on the left side in Ningxia was thicker than for the other two regions, indicating a greater probability of strong meteorological drought than for Shaanxi and Gansu.

### Probability distribution estimation of relative meteorological yield

According to the definition of the relative meteorological yield in 3.2.2, Equations (3) and (4) were used to obtain a sample of the relative meteorological yield of maize in Gansu, Ningxia, and Shaanxi between 1978 and 2016. Gansu is used as an example (see Appendix E).

**Figure 4:**
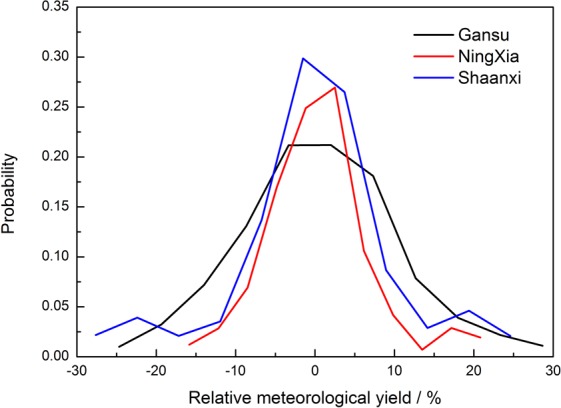
Probability of relative meteorological yield.

The probability distribution curve (Fig. 4) showed peaks of probability in Gansu, Ningxia, and Shaanxi at −3.33%, 2.50%, and −1.50%, respectively, with corresponding probability values of 0.21, 0.27, and 0.30. Skewness in Gansu, Ningxia, and Shaanxi was 0.63, 1.11, and 0.46, respectively; and corresponding kurtosis was −1.32, −0.23, and 0.25. The above data show that the distributions of the probability curves of relative meteorological yield in Gansu and Ningxia were right-biased and flatter than standard normal distribution; however, the curve in Shaanxi was left-biased and steeper than the standard normal distribution. When the relative meteorological yield was between −20% and −5% and between 5% and 18%, Gansu had the highest probability of occurrence, followed by Shaanxi and Ningxia. Thus, the probability distribution of relative meteorological yield for maize in Gansu was highly discrete, with thick tailings, large uncertainties, and more extreme values, which were strongly affected by meteorological conditions, followed by Shaanxi and Ningxia.

### Analysis of maize drought vulnerability

Using MDD as a hazard factor and relative meteorological yield as a result of drought risk, we obtained a causal sample of maize drought vulnerability and analyzed drought vulnerability of maize (see Appendix F). Because meteorological data are predictable, the relative meteorological yields corresponding to the weather conditions were obtained by bringing the predicted meteorological data into the information matrix *R*_*UxV*_, which can be used to forecast harvest to some extent.

**Figure 5:**
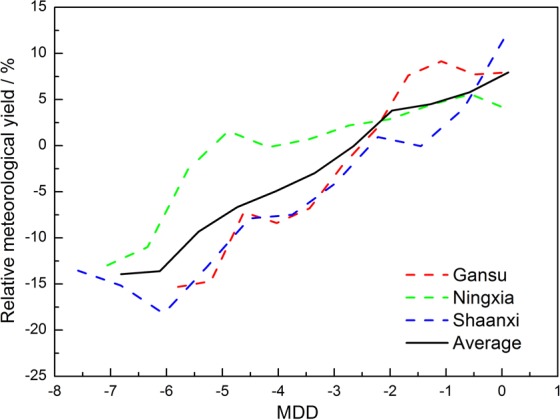
The vulnerability curve of meteorological drought degree vs. relative meteorological yield.

The relative meteorological yields of Gansu, Ningxia, and Shaanxi decreased with an increased MDD, and there were slight fluctuations due to disturbance of the data (Fig. 5). On average, when the MDD was greater than −2.60, the relative meteorological yield of maize increased within 10%; When the MDD was less than −2.60, the relative meteorological yield of maize decreased within 15%. For example, in 1998, the MDDs of Gansu, Ningxia and Shaanxi were −0.58, −0.71 and −0.38, respectively. This year was a mild drought year, consistent with the records of the “China Meteorological Disaster Canon”, while the relative meteorological yield increased by 13.33%, 5.78% and 19.67%, respectively. Regionally, when MDD exceeded −2.2, maize was most vulnerable to drought in Shaanxi followed by Ningxia and Gansu. When MDD was less than −2.2, maize was most vulnerable to drought in Shaanxi followed by Gansu and Ningxia.

### Maize drought risk

Substituting the probability distribution *P* of MDD and the vulnerability function *F*(*x*) obtained from the normal information diffusion method into Equations (5) and (6), the expected values of relative meteorological production in Gansu, Ningxia, and Shaanxi of 1.36%, 2.48%, and −1.76% were obtained, respectively. In other words, according to the previous precipitation data and production records, the relative meteorological yields in Gansu and Ningxia increased by 1.36% and 2.48% in one year, respectively, and for Shaanxi they were reduced by 1.76%. Thus, Shaanxi had the highest maize drought risk, followed by Gansu and Ningxia.

## Discussion

First, because natural disaster systems are very complex, it is difficult to assume the appropriate probability distribution type, so non-parametric estimation methods are important. The histogram method is the simplest non-parametric method for estimating probability distribution. Because of the scarcity of natural disaster information, sample sizes are usually very small. The traditional histogram method ignores the difference between different sample points which fall into the same histogram interval, and the result is only approximate. However, the method of information distribution and diffusion can improve the utilization rate of incomplete information and the accuracy of system recognition. Therefore, the risk value obtained by this method also considered the random uncertainty of meteorological factors and the nonlinearity of the vulnerability curve, and the results have high credibility. Liu *et al*.^38^ reported, for the same sample, that the information diffusion method was significantly better than the traditional histogram method, and the information diffusion technique has some practical reference value in research on natural disaster risk assessment for small sample.

Second, when the degree of meteorological drought was low, the relative meteorological yield of maize increased to some extent. The main reasons for this phenomenon are: (1) Mild drought stress can improve maize drought adaptability. For example, mild drought can increase the specific surface area of maize roots, increase the oxidation and reduction ability of roots, and improve maize drought resistance. At a later stage, once moisture conditions improve, maize can show a high over-compensatory effect and increased yield; with an increased MDD, the self-restoring capacity of maize decreased gradually after drought stress or is irreversibly damaged, resulting in yield reductions^58,59^. Qin and Wang^60^ found that root-induced cytokinin is a key factor in maize compensatory growth during post-drought rewatering. (2) Water sensitivity of maize differs at different growth stages. Liu *et al*.^61^ found that mild drought hardening at the seedling stage could result in higher antioxidant enzymes activities and removal of reactive oxygen species, and so reduce the degree of membrane lipid peroxidation, which would result in the photosynthetic efficiency producing a compensation effect after rewatering. (3). The recovery extent or growth limitation caused by previous drought strongly depends on the drought cycles and a full or partial recovery of growth may occur^62^.

Third, the statistical scale of maize unit yield available in the study is provincial. For consistency with the statistical scale of the data, the MDD was constructed using the average SPI3 values of all meteorological stations in each province. Comparing the results with the records of “China Meteorological Disaster Canon”, “Climate Impact Assessment”, and “China Meteorological Disaster Yearbook” showed that the MDD index basically reflected the actual drought situation in the study area. According to the records, the serious drought years in Gansu were 1982, 1991, 1995, 1997, 2000, 2001, and 2009; in Ningxia were 1980, 1982, 1987, 2000, 2005, 2008, and 2011; and in Shaanxi were 1995, 1997, 2000, 2001 and 2004. However, some errors may occur in some years, for example, the drought recorded in 1994 was severe, but the drought reflected by MDD was lighter. The main reasons for this phenomenon are as follows: (1). SPI represents meteorological drought, and the data recorded in the yearbook represents agricultural drought. When meteorological drought is transmitted to agricultural drought, it is affected by many factors, such as vulnerability and sensitivity of disaster-bearing bodies, and disaster prevention and mitigation capacity^63,64^. (2). The spatial frames applied here (i.e. province) is missing an adequate biological/ecological/climatological meaning. Therefore, information loss results from averaging of data not belonging to climatically similar locations. In our future work, we will use spatial frames such as climatic divisions, watersheds, or area clusters formed by grouping analysis of geographic information system (GIS) using relevant variables in gridded data form.

Fourth, it should be noted that the information diffusion method used in this paper was based on the normal diffusion function. This function reflects a uniform diffusion process, and there may be some asymmetric structure or irregularity among the elements of the incomplete sample obtained. For example, the irregular proportional relationship between variables needs to consider the diffusion speed and diffusion method in different directions. Therefore, the asymmetric diffusion of information should be considered in future vulnerability and risk analysis.

## Conclusions

According to the meteorological drought and relative meteorological yield, the drought vulnerability characteristics of maize were analyzed in Gansu, Ningxia, and Shaanxi in the eastern part of Northwest China. Information distribution and diffusion technology were introduced into the analysis of small sample events to obtain the probability distribution of the degree of meteorological drought and the relative meteorological yield in the study area. Specifically, the probability of moderate meteorological drought in Shaanxi was high, data dispersion in Gansu was relatively strong, and the probability of strong meteorological drought in Ningxia was high. The probability distribution of the relative meteorological yield of maize in Gansu had strong dispersion, with thick tailing, large uncertainties, and contained more extreme values, which were strongly affected by meteorological conditions. Based on the causal relationship of drought events in maize, the vulnerability curves of MDD vs. relative meteorological yield in Gansu, Ningxia, and Shaanxi were obtained, and showed that the relative meteorological yield decreased with an increased degree of meteorological drought. When the degree of meteorological drought was low, the relative meteorological yield of maize increased within 10%. Overall, drought vulnerability of maize in Shaanxi was the greatest, followed by Gansu and Ningxia – in accordance with the actual situation. According to the risk definition and equation, the drought risk for maize was obtained and was highest in Shaanxi followed by Gansu and Ningxia.

## Supplementary information

Appendix
