# Supplementary material for: Characteristics of drought vulnerability for maize in the eastern part of Northwest China
Source: Sci Rep. 2019 Jan 30;9:964. doi: 10.1038/s41598-018-37362-4 (PMC6353889; doi:10.1038/s41598-018-37362-4)
Supplement: Supplementary file 1 — Appendix [file 41598_2018_37362_MOESM1_ESM.pdf]

Characteristics of drought vulnerability for maize in the eastern part of Northwest China

Wang Ying, Zhao Wen, Zhang Qiang [*], Yao Yu-bi

Key Laboratory of Arid Climatic Change and Reducing Disaster of Gansu Province, Key Open Laboratory of Arid Change and Disaster Reduction of CMA, Institute of Arid Meteorology, China Meteorological Administration, Lanzhou, Gansu 730020, China

* Corresponding author: Qiang Zhang (zhangqiang@cma.gov.cn)

Postal address:

2070 East Donggang Road

Lanzhou Institute of Arid Meteorology,

China Meteorological Administration

Lanzhou 730020, Gansu Province

China

Email: wangyn924@163.com

Phone: +86-931-2402777

Fax: +86-931-4677529

Appendix A. Summary of equations to relative meteorological yield model parameters

This study used the method of linear moving average to separate trend yield; that is, changes in the time series of yield within a certain period of time were regarded as a linear function. With a continuous sliding of the period, a straight line constantly changes its position and slides backward to reflect the historical evolution of yield. The linear regression models for each time period were calculated sequentially, and the average value of sliding regression simulation for each linear function at each time point was calculated, which represents the trend output of maize in that year. If the linear regression equation of a certain stage is:

$$Y_i(t) = a_i + b_i t \qquad \text{(A1)}$$

where $i = n + k + 1$ is the number of equations, n is the number of sample sequences, and k is the time step. To eliminate the effect of short-cycle fluctuations, the time step was taken as 11. The t is the time serial number; when $I = 1$, $t = 1, 2, 3, \ldots, k$; $I = 2$, $t = 2, 3, 4, \ldots, k + 1$, and so on. The function value $Y_i(t)$ of each equation was calculated at t points, so that every t point had q function values. The mean value of the q function:

$$\overline{Y_j}(t) = \frac{1}{q}\sum_{j=1}^{q} Y_j(t) \qquad \text{(A2)}$$

Connecting $\overline{Y_j}(t)$ to each point indicates the historical evolution of production can indicate the trend yield.

Appendix B. Summary of equations to information distribution method

The information distribution method was used to obtain the probability distribution of samples. The sample set of observations for the study area in the past n years is termed X:

$$X = \{x_1, x_2, \ldots, x_n\} \quad \text{(B1)}$$

According to the maximum and minimum values of X, the appropriate step length Δ was selected to obtain the control point space U of the observation sample:

$$U = \{u_1, u_2, \ldots, u_n\} \quad \text{(B2)}$$

From the one-dimensional linear information distribution Equation (B3), the information carried by the sample point x_i in X is distributed to the corresponding control point space u_j by the amount q_{ij}:

$$q_{ij} = \begin{cases} \left(1 - \frac{|x_i - u_j|}{\Delta}\right), |x_i - u_j| \leq \Delta \\ 0, \text{others} \end{cases}; \text{ i = 1,2,…,n; j = 1,2,…, m} \quad \text{(B3)}$$

Let:

$$Q_j = \sum_{i=1}^{n} q_{ij} \quad \text{(B4)}$$

Q_j is the total distribution information on the control point u_j, thus a soft frequency histogram of X was obtained.

$$p_j = \frac{Q_j}{n} \quad \text{(B5)}$$

The probability distribution of each control point space can be obtained:

$$P = \{p(u_1), p(u_2), p(u_3), \ldots, p(u_m)\} = \{p_1, p_2, p_3, \ldots, p_m\} \quad \text{(B6)}$$

The P has high accuracy in estimating probability distributions of

observed samples.

Appendix C. Summary of equations to information diffusion method

The normal information diffusion method was used to identify vulnerability functions. Assuming that W is the data-set of the MDD x and the relative meteorological yield y, the causality is:

$$W = \{(x_1, y_1), (x_2, y_2), \ldots, (x_n, y_n)\} \quad \text{(C1)}$$

According to the requirements of the calculation accuracy, the step lengths Δx and Δy were selected to generate the input and output monitoring spaces, respectively:

$$U = \{u_1, u_2, \ldots, u_m\}; \quad \text{(C2)}$$

$$V = \{v_1, v_2, \ldots, v_t\}. \quad \text{(C3)}$$

Using the two-dimensional normal diffusion Equation (C4), the information carried by the sample points (x_i, y_i) in W is assigned to the points u_j and v_k in U and V by the amount μ_{ijk}. Any sample (x_i, y_i) in the set of samples X can be spread to the input and output universe U and V using the normal distribution rule:

$$\mu_{ijk} = \frac{1}{2\pi h_x h_y} exp\,[-\frac{(u_j - x_i)^2}{2h_x^2} - \frac{(v_k - y_i)^2}{2h_y^2}] \quad \text{(C4)}$$

The diffusion coefficient h_x in Equation (C4) is calculated using Equation (C5):

$$h_x = \begin{cases} 0.8146(b-a), n = 5; \\ 0.5690(b-a), n = 6; \\ 0.4560(b-a), n = 7; \\ 0.3860(b-a), n = 8; \\ 0.3362(b-a), n = 9; \\ 0.2986(b-a), n = 10; \\ 2.6851\,(b-a)/(n-1), n \geq 11. \end{cases} \tag{C5}$$

where $b = max_{1 \leq i \leq n}\{x_i\}$; $a = min_{1 \leq i \leq n}\{x_i\}$. Let:

$$Q_{jk} = \sum_{i=1}^{n} \mu_{ijk}, \tag{C6}$$

We obtained the original information matrix Q (Equation C7) with W on $U \times V$. Normalization of each column of Q was conducted to generate fuzzy relationship R (Equation C8), which describes the fuzzy relationship between MDD and relative meteorological yield of maize. Using a fuzzy approximation reasoning model, we derived the output y_0 from the input x_0 to produce a vulnerability function.

$$Q = \begin{pmatrix} Q_{11} & \cdots & Q_{1t} \\ \vdots & \ddots & \vdots \\ Q_{m1} & \cdots & Q_{mt} \end{pmatrix} \tag{C7}$$

$$\begin{cases} R = \{r_{jk}\}_{m \times t;} \\ r_{jk} = Q_{jk}/s_{k;} \\ s_k = max_{1 \leq j \leq m}\, Q_{jk.} \end{cases} \tag{C8}$$

If the distinct input value x_0 (hypothetical) is known, an input fuzzy set $\tilde{x}_0$ can be obtained using the information distribution Equation (C9):

$$\mu_{x_0}(u_j) = \begin{cases} 1 - \frac{|x_0 - u_j|}{\Delta}, |x_0 - u_j| \ll \Delta; \\ 0, \quad others. \end{cases} \tag{C9}$$

where $\Delta = u_{j+1} - u_j$. We used Equation (C10) to derive the output value $\tilde{y}_0$ from x_0 and R.

$$\mu_{y_0}(v_k) = \sum_{1 \leq j \leq m} \mu_{x_0}(u_j) \times r_{jk}\,, k = 1,2, \ldots, t. \tag{C10}$$

To bring the fuzzy set $\tilde{y}_0$ into Equation (C11), we can defuzzify it to a certain value y_0:

$$y_0 = \frac{\sum_{1 \le k \le t} \mu_{y_0}(v_k) \times v_k}{\sum_{1 \le k \le t} \mu_{y_0}(v_k)} \quad \text{(C11)}$$

Under small sample conditions, $F(x_0) = y_0$ can better reflect the causality between X and Y in Equation (C1), which to some extent solves the problem of insufficient sample size.

Appendix D. Probability distribution estimation of MDD

$$X = \{x_1, x_2, \ldots, x_n\} = \{-1.25, -3.17, \ldots, -1.04\}, \quad \text{(D1)}$$

To determine the number of traditional histogram intervals m according to the histogram progressive optimization interval number Equation (D2):

$$m = 1.87(n-1)^{2/5}, \quad \text{(D2)}$$

where n is the length of the sample, i.e., n = 39. Thus m = 8.01, which was divided into nine intervals in this research. Because the data range of MDD was –5.42 to –0.22, the starting point was –5.50, step length was 0.59, and change range was –5.50 to –0.20. To change the distinct boundary of the traditional histogram into the fuzzy boundary of the information distribution method, the midpoint of the foregoing interval was used as the basic control point for information distribution. Simultaneously, because the total range of information distribution exceeded the range of the traditional histogram, a control point was

extended outwardly at both ends of the basic control point. The control point space U is shown in Equation (D3):

$$U = \{-5.79, -5.21, -4.62, -5.03, -3.44, -2.85, -2.26, -1.67, -1.08, -0.49, 0.09\}; \quad \text{(D3)}$$

Information distribution Equation (B3) was used to assign information X in Equation (D1) to the control point space U of Equation (D3), and the information assigned to each point was processed using Equations (B5) and (B6). The probability distribution map of MDD is shown in Equation (D4):

$$P = \{p(u_1), p(u_2), p(u_3), \dots, p(u_m)\} = \{0.016, 0.033, 0.036, \dots, 0.018\} \quad \text{(D4)}$$

Appendix E. Probability distribution estimation of relative meteorological yield

$$Y = \{y_1, y_2, \dots, y_n\} = \{25.54, 11.12, \dots, -4.66\}, \quad \text{(E1)}$$

According to Equation (D2), the sample was divided into nine intervals and the sample data range was –21.14 to 25.54. The starting point was –22, step length was 5.33, and the change range was –22 to 26. The midpoint of the above interval was used as the basic control point for information distribution, and a control point was extended outwardly at both ends of the basic control point to obtain the fuzzy boundary control point space V shown in Equation (E2):

$V = \{-24.67, -19.33,\ -14.00,\ -8.67,\ -3.33,\ 2.00,\ 7.33,\ 12.67,$
$18.00,\ 23.33,\ 28.67\}$ (E2)

Information distribution Equation (B3) was used to assign information *Y* in Equation (E1) to the control point space *V* of Equation (E2), and the information assigned to each point was processed using Equations (B5) and (B6). The probability distribution map of relative meteorological yield is shown in Equation (E3).

$P = \{p(v_1),\ p(v_2),\ p(v_3),\ \dots,\ p(v_t)\} = \{0.010,\ 0.032,\ 0.072,$
$\dots,\ 0.011\}$ (E3)

Appendix F. Analysis of maize drought vulnerability

$W = \{(x_1, y_1), (x_2, y_2), \dots, (x_n, y_n)\} =$
$\{(-1.25, 25.54), (-3.17, 11.12), \dots, (-1.04, -4.66)\}$ (F1)

In order to distinguish from the notation of the control point space in Equation (D3), subscripted capital letters are used to mark the control point space used in information diffusion. U_{spi} is the input control point space for MDD and V_{yield} is the output control point space for relative meteorological yield. In theory, the control points for normal diffusion should be as many as possible, but they are usually selected on the accuracy of the observation points. Too many control points have little significance for improving recognition accuracy and increase the amount of calculation[30]. Therefore, the step length of the input control point space

U_{spi} was set to 0.15, the starting point to –5.79, and the range of change of –5.79 to 0.09, with a total of 41 control points (Equation F2). The number of output control points was consistent with U_{spi}, and V_{yield} had a step length of 1.33 with a range of change of –24.67 to 28.67 (Equation F3).

$$U_{spi} = \{-5.79, -5.65, -5.50, \ldots, 0.09\}; \quad \text{(F2)}$$

$$V_{yield} = \{-24.67, -23.33, -22.00, \ldots, 28.67\}; \quad \text{(F3)}$$

Then, according to Equations (C4)–(C7), we obtained the information matrix $Q_{U\times V}$, which reflects the causality between MDD and relative meteorological yield. According to Equation (C8), $Q_{U\times V}$ was normalized to obtain a normalized information matrix $R_{U\times V}$, and x_0 was set as the sample in Equation (D1). The fuzzy set on U_{spi} was obtained by the information diffusion method, and the fuzzy set on V_{yield} by Equation (C10). Then the distinct output value was obtained using Equation (C11).
